# Assessment of Tail-Cutting in Frozen Albacore (*Thunnus alalunga*) Through Ultrasound Inspection and Chemical Analysis

**DOI:** 10.3390/foods13233860

**Published:** 2024-11-29

**Authors:** Masafumi Yagi, Akira Sakai, Suguru Yasutomi, Kanata Suzuki, Hiroki Kashikura, Keiichi Goto

**Affiliations:** 1 School of Marine Science and Technology, Tokai University, 3-20-1 Orido, Shimizu-ku, Shizuoka-shi 424-8610, Shizuoka, Japan; 2 Artificial Intelligence Laboratory, Fujitsu Limited, 4-1-1 Kamikodanaka, Nakahara-ku, Kawasaki-shi 211-8588, Kanagawa, Japan; 3 Graduate School of Marine Science and Technology, Tokai University, 3-20-1 Orido, Shimizu-ku, Shizuoka-shi 424-8610, Shizuoka, Japan

**Keywords:** frozen tuna, frozen albacore, tail-cutting, fat content, ultrasound, machine learning

## Abstract

Fat content is the main criterion for evaluating albacore quality. However, no reports exist on the accuracy of the tail-cutting method, a method used to assess the fat content of albacore. Here, we evaluated this method by comparing it with chemical analysis and ultrasound inspection. We measured the actual fat content in albacore using chemical analysis and compared the results with those obtained using the tail-cutting method. Significant discrepancies (99% CI, *t*-test) were observed in fat content among the tail-cutting samples. Using chemical analysis as the ground truth, the accuracy of tail-cutting from two different companies was 70.0% for company A and 51.9% for company B. An ultrasound inspection revealed that a higher fat content reduced the amplitude of ultrasound signals with statistical significance (99% CI, *t*-test). Finally, machine learning algorithms were used to enforce the ultrasound inspection. The best combination of ultrasound inspection and a machine learning algorithm achieved an 84.2% accuracy for selecting fat-rich albacore, which is better than tail-cutting (73.6%). Our findings suggested that ultrasound inspection could be a valuable and non-destructive method for estimating the fat content of albacore, achieving better accuracy than the traditional tail-cutting method.

## 1. Introduction

Tuna is heavily fished across the globe. Their meat is valued for its richness in essential nutrients such as proteins, fats, and vitamins [1,2,3,4,5,6]. The fat contents of tuna meat consist of highly unsaturated fatty acids such as docosahexaenoic acid and eicosapentaenoic acid [7], and the fatty acids from tuna play an important role in preventing the decline in serum lipids and maintaining cognitive function. The global production of tuna has increased from less than 3 million tons in 1990 to more than 5 million tons in 2022 [8]. The number of countries fishing for tuna species exceeded 100 in 2022, and the countries that produce close to 100,000 tons are Indonesia (546,974 tons), Taiwan (146,830 tons), Japan (141,119 tons), Spain (128,530 tons), Mexico (127,904 tons), Oman (104,043 tons), and Ecuador (93,565 tons) [8]. Albacore (*Thunnus alalunga*) has a more stable price and a larger catch (247,000 tons/year) than other types of tuna [9,10,11,12,13,14,15,16,17,18]. As such, the market for frozen albacore is large. Most albacore is used for canning and sashimi; however, they are often caught via purse seine fishing, which results in varying quality [19]. There are a few general studies evaluating the criteria for tuna quality [20,21,22,23]. While evaluating the freshness of tuna is very important for determining its quality, for albacore, fat content is the main and only criterion currently employed for quality evaluation and it has a significant impact on price. As fat content affects the price of frozen albacore, highly accurate inspection technology is required. In this study, we focused on fat content and investigated the inspection methods based on multiple analysis perspectives.

Tail-cutting is a traditional method that is widely used to assess the fat content of albacore. In this method, skilled experts cut the tails of frozen albacore, thaw them in warm water, and visually and subjectively rank them by observing the cut surface [24,25,26,27]. Despite its widespread use, tail-cutting has some challenges, such as the small cross-section samples and the difficulty in distinguishing fat from pale-colored meat. Additionally, tail-cutting is a destructive method that potentially damages the tuna [28]. Tail-cutting results in food loss; the shelf life is reduced when more of the fish is uncovered [29,30,31]. Furthermore, the tail-cutting process is difficult in that it relies on a limited number of experts. There is also a marketing problem in tail-cutting. There are two markets for tuna: those for round fish and fillets. Once the fish have been tail-cut, they cannot be sold as round fish. Moreover, there have been no previous reports on the accuracy of the tail-cutting method; clarifying this method is therefore one of the purposes of this study.

Although traditional methods are the mainstream in the seafood industry, in the rest of the food industry, non-destructive quality-assessment techniques, such as the near-infrared technique, are increasingly being considered, and researchers have been studying their use in many food fields [28,32,33]. Non-destructive techniques exist, such as the near-infrared technique. However, it has been reported that the thickness of the skin of large frozen tuna and the ice covering the surface makes it difficult to evaluate them using near-infrared spectroscopy, in practice [34,35]. On the other hand, machine learning, an algorithm that recognizes correct patterns by learning from data [36,37,38,39,40,41,42,43,44], is attracting attention as an automated inspection method because it can build classification models without manual design object features. Medeiros et al. performed ensemble learning using multiple classification models with color values obtained by preprocessing digital images, demonstrating the possibility of estimating raw tuna and salmon freshness [45]. Several recent studies have used ultrasound as an input for machine learning models to inspect inside (e.g., freshness) the target objects [46,47]. Tokunaga et al. proposed a method to estimate the fat content and texture of raw fish from ultrasound signals using self-organizing maps and radial basis functions [46]. Furthermore, Sakai et al. proposed a machine learning method using multiple ultrasound signals for frozen tuna, demonstrating the possibility of estimating freshness with high accuracy [47]. Machine learning methods using ultrasound are expected to serve as an alternative to the traditional tail-cutting method and several studies [46,47] have been published in this regard. However, no previous studies have investigated using ultrasound to estimate the fat content of frozen albacore.

Based on the above background, this study focused on the fat content of albacore and investigated the inspection methods based on multiple analysis perspectives. More specifically, we clarified the effectiveness of the empirically accepted tail-cutting method by comparing it with two other methods: chemical analysis and ultrasound inspection. This is the world’s first attempt to compare fish fat content inspection methods. We prepared 57 frozen albacore samples that were assessed using tail-cutting from two Japanese tuna trading companies. In the first step, we chemically analyzed the samples to quantitatively evaluate the inspection methods. In the next step, we acquired ultrasound signals and compared them with the chemical analysis results to investigate the signal trends. In the final step, we trained machine learning models with ultrasound signals to examine whether they can become a new non-destructive inspection method for estimating the fat content of frozen albacore.

### Related Work

Although the relationship between tuna quality and market value is important in the industry, there is limited academic research on this topic. One pioneering study was conducted by Shimose et al. [48]. They investigated the relationship between market price and fat content for 150 bluefin tuna (*Thunnus orientalis*), and found that tuna with more fat content traded at a higher price. Fat content positively influences the bid price of both Pacific bluefin tuna and bigeye tuna (*Thunnus obesus*) [49].

There are several candidates for the non-destructive inspection of tuna. X-ray and radiation methods [50,51,52] are harmful and expensive and, therefore, inappropriate for practical use. The electric current method [53,54,55] cannot evaluate the interior of frozen tuna because most of the current passes along the surface due to the presence of water droplets. Inspection based on infrared light [56,57,58] is also popular. However, it has been reported that inspecting frozen tuna using infrared spectroscopy is difficult due to the glazed frost and thick skin. Ultrasound is a proven method for raw fish [46,59,60,61,62,63] and the non-destructive inspection method for frozen fish is commonly reported [47]. For these reasons, we chose ultrasound as our non-destructive inspection method.

## 2. Materials and Methods

**Figure 1:**
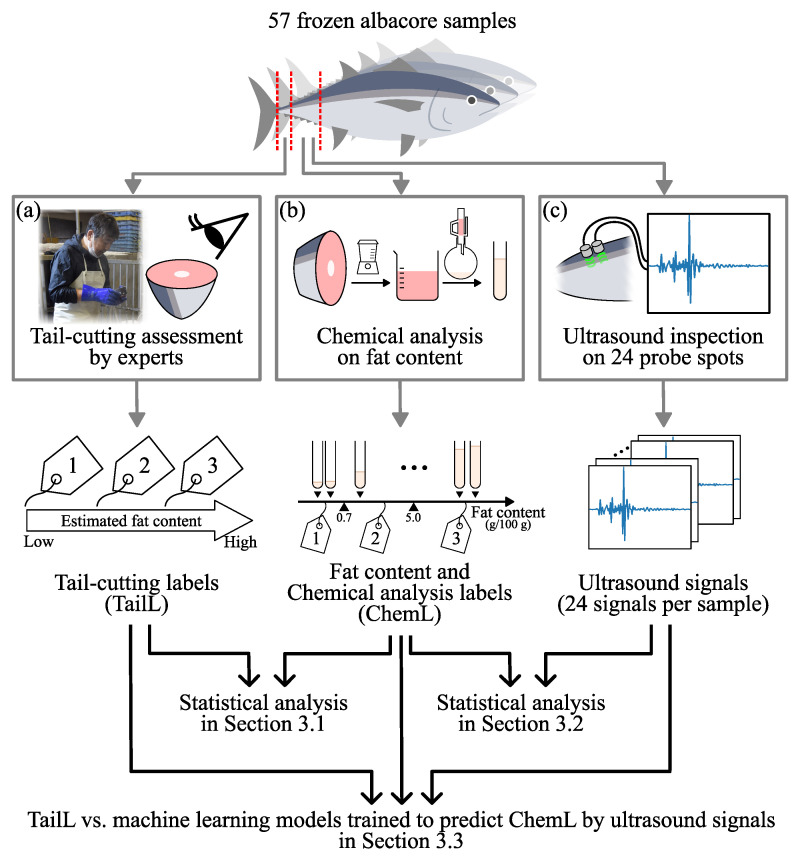
Overview of this study. We used three inspection methods: (**a**) tail-cutting, (**b**) chemical analysis, and (**c**) ultrasound inspection to estimate the fat content in frozen albacore. In addition, we systematically evaluated the inspection methods by comparing the data or labels obtained from each analysis process in Section 3.1, Section 3.2, and Section 3.3, respectively. Labels 1, 2, and 3 are defined in Section 2.2 and Section 2.3, respectively.

Figure 1 presents the overall framework of this study, which involved the acquisition and statistical evaluation of data from 57 frozen albacore samples using tail-cutting, chemical analysis, and ultrasound signals. Each independent analysis process (Figure 1a–c) was applied to the same sample. We systematically evaluated the inspection methods for frozen albacore by comparing the data or labels obtained from each analysis process (see Section 3.1, Section 3.2 and Section 3.3).

### 2.1. Samples

**Figure 2:**
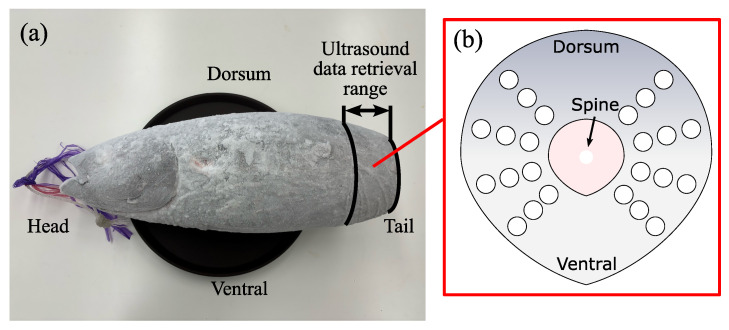
(**a**) Example of the frozen albacore used in this study and (**b**) an illustration of a caudal part of the sample viewed from the tail-side. White markers (◯) indicate the probe spots for ultrasound inspection.

A total of 57 frozen albacore samples were obtained from companies A and B. Both companies have leading market shares in the fisheries industry of the country and are known for their reliable quality. Each sample was caught in the Atlantic or Indian Ocean, half in July–September and the other half in May. Figure 2a displays an example albacore sample. The samples were cut off 10 cm from the tail, and individually sealed in plastic bags, vacuum-sealed, and stored at −60°C. Detailed sample data and individual information are provided in Table A1.

### 2.2. Tail-Cutting

Each sample was visually assessed for fat content via the tail-cutting method (Figure 1a). The process carried out by experts at these companies involved the following:

1. Cutting approximately 5 cm from the tail side of the frozen albacore using an electric saw.
2. Thawing the cut surface by immersing the caudal fragment in warm water.
3. Visually assessing the fat content based on the muscle color on the thawed caudal surface.

We assigned three labels to describe fat content based on tail-cutting, referred to as TailL (tail-cutting label), and categorized as 1, 2, and 3. The larger the TailL number, the higher the fat content of the sample. The criteria for TailL are based on the experience of experts at each seafood company. To avoid data variability, samples were prepared so that the number of each label was equal. The information on the size and weight of samples is provided in Table 1. It was found that the larger the TailL, the shorter the body length and the lighter the weight.

### 2.3. Chemical Analysis

In this study, chemical analysis was performed to quantitatively evaluate the inspection methods (Figure 1b). The Soxhlet extraction method [64] was used for determining fat content. This method is applied to easily crushable foods with high fat content not coupled with the tissue component. The solvent used was diethyl ether, and the number of samples was 2. The following processes were carried out.

1 A site approximately 10 cm from the caudal side of all 57 albacore samples was crushed and homogenized. The samples were preserved and frozen until analysis.
2 To dehydrate each sample, W=3.0 g of the crushed sample was collected. The collected samples were dehydrated by adding 2.5 g of sodium sulfate (anhydrous) and then transferred to a cylindrical filter paper.
3 To control the weight error of the flasks, they were pre-dried in an electric constant temperature dryer at 100–105 °C for 2 h. Afterward, the flask was transferred to a desiccator and weighed after 1 h of cooling ($W_{0}$ g).
4 To extract the fat content from each sample, we placed approximately 100 mL of diethyl ether in the flask. Extraction was performed in an electric thermostatic bath at 50 °C for 8–16 h.
5 After extraction, samples were dried in an electric constant temperature dryer at 100–105 °C for 1 h. Thereafter, the flask was transferred to a desiccator and weighed after 1 h of cooling ($W_{1}$ g).

The fat content in the sample was calculated using the following equation using three weight values (*W*, $W_{0}$, and $W_{1}$) obtained in the above procedure.

(1) $$\begin{array}{c} \mathrm{fat}\;\mathrm{content}\;[g/100\;g]=\frac{\left(W_{1}-W_{0}\right)}{W}\times 100 \end{array}$$

The fat content from the above chemical analysis represents the mass per 100 g of the edible portion.

Labels derived from chemical analysis were designated as ChemL (chemical analysis label) = 1, 2, and 3. They were assigned as follows: a ChemL of 1 indicates fat content less than 0.7 g, 2 indicates fat content between 0.7 and 5.0 g, and 3 indicates fat content greater than 5.0 g. The threshold values were based on the Fatty Acid Composition Table from the Standard Tables of Food Composition in Japan (8th Revised Edition), Updated and Enlarged Version 2023 [65]. In the experiments described in the next section, we compare ChemL with TailL and ultrasonic signals to investigate the validity of each inspection method.

### 2.4. Ultrasound Inspection

#### 2.4.1. Ultrasound Signals

We performed an ultrasound inspection—a non-destructive inspection method—to estimate the fat content in frozen albacore and compared it with tail-cutting (Figure 1c). We collected ultrasound signals from 57 frozen albacore samples using the two-probe reflection method. This method uses one for transmission and another for reception. The distance between the two single-element ultrasonic transducers was 3 mm. We used glycerol and a 3 mm-thick silicon sheet with a hardness of 30 A as ultrasonic couplants. They were placed between the surface of the sample and both ultrasonic transducers. In our inspection procedure, we used the JPR-600C model ultrasonic pulser receiver and single-element composite ultrasonic transducers B0.5K20N with a center frequency of 500 kHz, produced by Japan Probe Company Ltd. (Yokohama City, Japan). Table 2 shows the ultrasonic experimental conditions used in this study.

The detailed positions where the data were collected are shown in Figure 2b. Based on a previous study [47], ultrasound signals were collected from a cross-section 40–60 cm around the tail of the sample. Each sample was examined at 24 probe spots by two examiners, resulting in 48 signals per sample. In total, 2736 signals were acquired. The inspection process was completed within 40 min to prevent the frozen samples from thawing.

#### 2.4.2. Machine Learning with Ultrasound Signals

Inspired by related studies [46,47], we employed typical machine learning models using the acquired ultrasound signals. In the preprocessing phase, ultrasound signals were trimmed between 20 and 80 μs to focus on the largest reflections, typically emanating from the albacore’s spine. We utilized eight machine learning algorithms for classification: linear support vector machine (SVM [66,67,68,69]), radial basis function (RBF) SVM [70,71,72,73], linear discriminant analysis (LDA [74,75,76]), quadratic discriminant analysis (QDA [77,78,79]), random forests [80,81,82], adaptive boosting (AdaBoost [83,84,85]), multilayer perceptron (MLP [86,87,88]), and *k*-nearest neighbors (*k*-NN [89,90,91,92,93]). The SVMs used hinge loss and L2 penalty, and their cost parameter was set to 1.0. For the random forest, the maximum number of estimators was 100, and the maximum depth of the decision tree was unlimited. For the AdaBoost, the maximum number of estimators was 50, the maximum depth of the decision tree was 1, and the learning rate was 1.0. For the MLP, there were 3 layers, and the number of neurons in the middle layer was 100. We used Adam as an optimization method. For the *k*-NN, the number of neighbors for each sample point was 5, and the metric was Euclidean distance. We decided on the above hyperparameters based on the default values in Scikit-learn library [94].

We trained and tested all machine learning models to predict the ChemL obtained from chemical analysis. For simplicity, ChemL was converted into binary categories: (a) ChemL = 1 versus ChemL = 2, 3 and (b) ChemL = 1, 2 versus ChemL = 3. Scikit-learn’s default hyperparameters were used. In the inference phase, to reduce the effect of noise contained in the ultrasound signals, we integrated multiple signals obtained from the same albacore sample [47]. The machine learning model produced output class probabilities from each of the 24 signals and finally averaged and scaled them to 0–1. Subsequently, the model predicted the final class probabilities for ChemL.

### 2.5. Evaluation Metrics

#### 2.5.1. Tail-Cutting

In the experiment in Section 3.1, TailL estimated by tail-cutting was evaluated based on the fat contents and ChemL obtained by chemical analysis. We calculated the mean and standard deviation of fat content values within each TailL category. The accuracy of TailL was determined by using ChemL as the ground truth.

#### 2.5.2. Ultrasound Signals

In the experiment in Section 3.2, the ultrasound signals were evaluated using the ChemL obtained via chemical analysis. We computed the mean amplitudes of the ultrasound signals for each ChemL group and conducted a visual assessment. Additionally, we calculated the mean amplitudes over time for signals between 40 and 60 μs.

#### 2.5.3. Machine Learning with Ultrasound Signals

**Figure 3:**
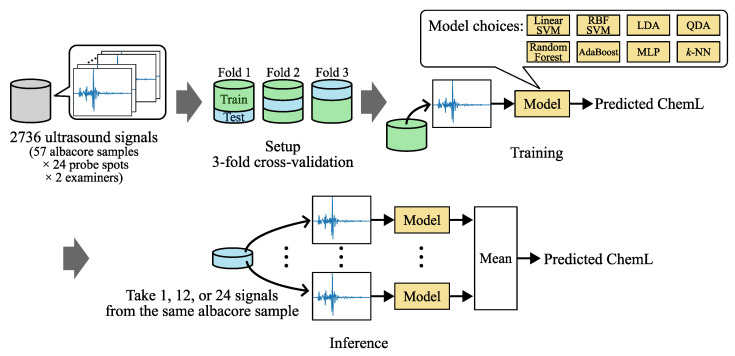
Overview of the process of machine learning with ultrasound signals. A total of 2736 ultrasound signals from 57 albacore samples were used in a 3-fold cross-validation setup to train eight machine learning models. During inference, multiple signals from the same albacore sample were averaged to predict the actual fat content (ChemL).

In the experiment in Section 3.3, to compare tail-cutting with ultrasound inspection, machine learning algorithms were employed that use ultrasound signals as input and predict fat content. The machine learning algorithms were validated using 3-fold cross-validation. Care was taken to ensure that the ultrasound signals from the samples did not overlap between folds. The mean accuracy across the folds was calculated. For models integrating multiple ultrasound signals, we utilized 1, 12, and 24 signals obtained from the same sample to compute the class probabilities for ChemL. The overall process is summarized in Figure 3.

### 2.6. Statistical Analysis

In the experiment in Section 3.1, we employed the *t*-test to assess the stastical significance between TailL groups [95]. Moreover, in the experiment in Section 3.2, we used the same test to assess the statistical significance of the mean amplitudes across the ChemL groups.

## 3. Results and Discussion

### 3.1. Relationships Between Tail-Cutting and Chemical Analysis

**Figure A1:**
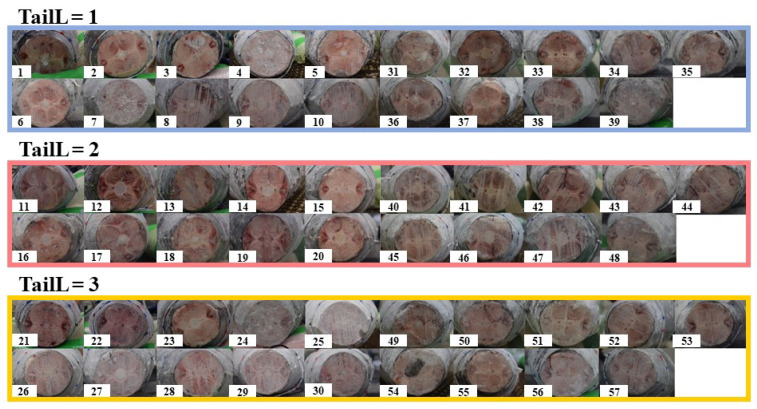
Cut surfaces of albacore after tail-cutting and tail-cutting labels (TailL) that show the result of estimated fat content by tail-cutting. Labels 1, 2, and 3 are defined in Section 2.2. The lower left number of each image indicates the serial of sample.

First, we investigated the validity of tail-cutting, which is a subjective method, using quantitative data from chemical analysis. The tail-cutting surfaces of the 57 samples are shown in Figure A1. As shown in the figure, distinguishing fat from fish meat is challenging due to their similar coloration; the flesh color typically ranges from white to pink, whereas fat appears white. Thus, only skilled experts can accurately carry out this process.

**Figure 4:**
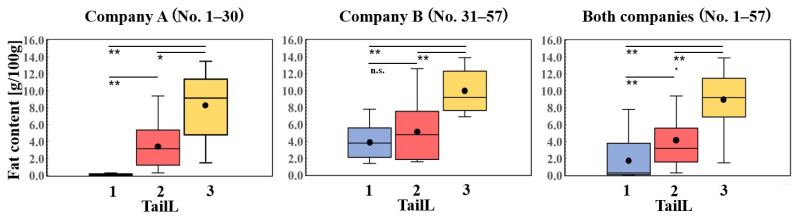
Box plots of the fat content obtained via chemical analysis: company A, company B, and both companies. The vertical axis shows the fat content value and the horizontal axis shows TailL (tail-cutting label). Circle marks (•) show the mean corresponding to each TailL. Horizontal lines in the box show median. The small dots shows the outliers corresponding to each TailL. Asterisks (*) and (**) show statistical significance at the 95% and 99% confidence interval, respectively. The symbol n.s. shows a non-significant difference at the 95% confidence interval.

Figure 4 shows the fat content values corresponding to each TailL. Statistics of the fat content of each TailL are shown in Table A2. The three boxplots represent data from company A, company B, and a combined analysis of both. The results indicate that fat content assessed by tail-cutting tends to increase in the value of TailL. The differences in fat content were statistically significant at the 99% confidence interval, except for TailL = 2 versus 3 in company A and TailL = 1 versus 2 in company B. In terms of albacore fat content, we consider that these results support the validity of tail-cutting as an assessment method. Figure 4 shows the fat content value differences in tail-cutting results between companies, especially at TailL = 1.

Furthermore, to examine the accuracy of tail-cutting for each company, we show the confusion matrix of each company in Table 3. The accuracy of TailL was evaluated using ChemL as the ground truth. We found that the accuracy was 70.0% for company A, 51.9% for company B, and 61.4% for both companies combined. TailL of company A tended to confuse ChemL = 2 and 3. In contrast, TailL of company B misclassified ChemL = 1. Combined with Figure 4, we can see the differences in tail-cutting criteria between the companies. For example, in company A, the mean fat content value at TailL = 1 was 0.11, whereas for company B, it was 4.02. This result suggests that the tail-cutting criteria depended on the policy that a limited number of experts implicitly decided. As the market and business environment affect the criteria for tail-cutting, it is important to establish an objective inspection method.

### 3.2. Relationships Between Ultrasound Signals and Chemical Analysis

**Figure 5:**
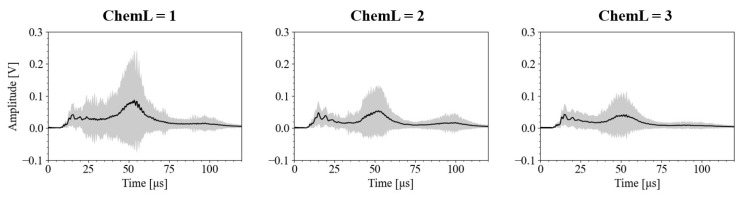
Mean amplitudes of ultrasound signals of each ChemL (chemical analysis label). The vertical axis shows amplitude [V], and horizontal axis shows elapsed time [μs]. Solid line shows mean amplitude and shadowed area shows standard deviation.

**Figure 6:**
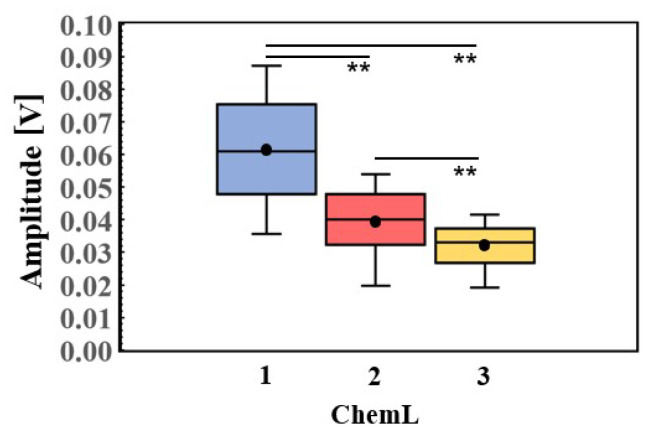
Box plots of the mean amplitude of ultrasound signals (from 40 to 60 μs) for each ChemL (chemical analysis label). The vertical axis shows amplitude [V] and the horizontal axis shows ChemL. Circle marks (•) show the mean corresponding to each ChemL. Horizontal lines in the box show median. Asterisks (**) show statistical significance at the 99% confidence interval.

To determine whether the physical quantities in the ultrasound signals obtained from the frozen albacore samples accurately reflect the fat content, we compared them with the chemical analysis results. Figure 5 presents the mean amplitude of the ultrasound signals for each ChemL, showing considerable variation around the 40–60 μs range. Figure 6 provides a boxplot of these mean amplitudes. The mean amplitudes for each level of fat content were statistically different with a 99% confidence interval. The mean amplitudes were 0.032 V for ChemL = 3, 0.039 V for ChemL = 2, and 0.061 V for ChemL = 1, indicating that ultrasound attenuation increases with fat content. These results showed that a higher fat content reduced the amplitude of ultrasound signals, indicating that ultrasound could differentiate levels of fat content.

### 3.3. Comparison of Tail-Cutting and Ultrasound Machine Learning Algorithms

Finally, we constructed machine learning models with ultrasound signals and evaluated whether ultrasound inspection can be an alternative method to tail-cutting in determining the fat content of frozen albacore. Table 4 presents the mean accuracy of eight different machine learning algorithms compared to that of the traditional tail-cutting method. Note that because this experiment performed binary classification, the accuracy of tail-cutting was different from that of the previous result (both companies in Table 3). In the scenario where ChemL = 1 versus ChemL = 2 and 3, tail-cutting had a mean accuracy of 73.6%, whereas the highest mean accuracy among the machine learning algorithms remained 84.2% using the same random forest approach with 24 signals (Table 4(a)). For the category ChemL = 1 and 2 versus ChemL = 3, the mean accuracy of tail-cutting was recorded at 78.9%. The highest mean accuracy among the machine learning algorithms was 84.2%, and this was achieved by a random forest algorithm using 24 signals (Table 4(b)). These results demonstrate that the machine learning algorithm utilizing ultrasound signals could match the mean tail-cutting accuracy. Additionally, the accuracy of the machine learning algorithms generally improved with increased signals in the inference process, corroborating the findings of previous studies [47]. Machine learning approaches generally work better with large teaching data, indicating that our methods could be further improved by acquiring more signals. Consequently, it is suggested that ultrasound inspection could be a valuable non-destructive method for estimating albacore fat content.

### 3.4. Further Discussion and Future Direction

This study aimed to evaluate the effectiveness of the traditional tail-cutting method for assessing albacore fat content and to explore alternative, non-destructive approaches. Our findings reveal significant limitations in the accuracy of tail-cutting, highlighting the need for more objective assessment techniques. While tail-cutting showed a general trend of increasing fat contents with higher TailL values, significant discrepancies were observed within TailL categories, particularly between the two companies involved in this study. This variability, with accuracies of 70.0% and 51.9% for companies A and B, respectively (using chemical analysis as ground truth), underscores the subjective nature of tail-cutting and the influence of implicit criteria employed by individual experts. This subjectivity introduces inconsistencies in quality assessment and pricing, emphasizing the importance of developing a standardized, objective method. Our results highlight the limitations of destructive and subjective quality assessment methods in the seafood industry. While tail-cutting remains prevalent in this sector, other food industries are increasingly adopting non-destructive techniques [32], further supporting the need for a shift in the seafood industry.

It is widely known that fatty cells become larger as the fat content in the tissues increase [96,97]. The ultrasound attenuation coefficient is affected by the size of the grains contained in the material; i.e., the larger the cell size, the more the ultrasound signals are attenuated [98,99]. This phenomenon has been widely observed in the medical field. The fact that high fat content leads to ultrasound attenuation is particularly taken advantage of for the examination of fatty liver [100,101]. Its application is expanding to other fields, e.g., meat fat content inspection [102,103]. This study demonstrated that the same phenomenon can be observed in frozen tuna.

We will now discuss the benefits of using ultrasound over tail-cutting. The former can be automated by mechanization [104] to improve the efficiency of quality assessments in on-site applications at fisheries, trading companies, and fishing ports. Such mechanization is expected to lead to uniform grading standards in the tuna market. One challenge in achieving mechanization is the development of robotic technology to perform ultrasound inspection automatically. While this study focused on the meat fat content, localized defects, such as thrombus, are also important in assessing fish quality. Regarding further future work, the sensory inspection [23,105,106,107,108,109] of tuna may provide more direct information on fish value.

## 4. Conclusions

In this study, we employed ultrasound inspection and chemical analysis to evaluate the effectiveness of the traditional tail-cutting method for assessing fat content in albacore. The insight of this study is as follows: (1) there is room for improving the accuracy of tail-cutting; (2) ultrasound inspection, an innovative method, performs better than tail-cutting. A larger sample size may be needed to obtain more accurate results and it will be a future study. Our study suggests ultrasound inspection as a potential alternative to tail-cutting and presents an opportunity to bring innovation to the tuna industry. The proposed approach is based on the manual inspection of individual albacore, which may be inappropriate in locations such as fishery trading companies and fishing ports. Consequently, constructing an automatic batch inspection method by mechanization is crucial in future work. 

## Figures and Tables

**Table 1:** Mean and standard deviation of size and weight of frozen albacore samples.

	Company A (No. 1–30)			Company B (No. 31–57)		
	TailL = 1	TailL = 2	TailL = 3	TailL = 1	TailL = 2	TailL = 3
	(N = 10)	(N = 10)	(N = 10)	(N = 9)	(N = 9)	(N = 9)
Body length [cm]	74.2 ± 6.7	68.7 ± 4.1	67.2 ± 4.7	67.3 ± 2.9	69.9 ± 3.5	66.4 ± 1.3
Weight [kg]	18.3 ± 3.1	13.2 ± 1.4	14.6 ± 2.9	13.7 ± 0.9	14.3 ± 1.9	12.9 ± 1.3
Ventral length [cm]	11.2 ± 1.5	12.3 ± 1.7	12.2 ± 1.7	12.7 ± 1.3	12.1 ± 1.1	13.8 ± 2.1
Dorsal length [cm]	10.6 ± 2.0	12.5 ± 1.7	12.0 ± 1.4	11.6 ± 0.5	12.8 ± 0.7	13.3 ± 2.2
Right length [cm]	10.2 ± 1.3	12.2 ± 1.6	12.1 ± 1.6	12.6 ± 1.5	11.8 ± 0.9	12.9 ± 1.8
Left length [cm]	10.4 ± 1.3	12.5 ± 1.8	11.8 ± 2.1	12.1 ± 1.5	12.3 ± 1.1	13.4 ± 2.2

**Table 2:** Ultrasonic experimental conditions used in this study.

Conditions	
Input voltage	100 V
Damping resistance	100 Ω
Gain	40 dB
Acquired signals	100 kHz high-pass filter
Sampling frequency	50 MHz

**Table 3:** Confusion matrix of tail-cutting. Here, labels derived from chemical analysis were designated as ChemL (chemical analysis label) = 1, 2, and 3. They were assigned as follows: a ChemL of 1 indicated a fat content less than 0.7 g, 2 indicated a fat content between 0.7 and 5.0 g, and 3 indicated a fat content greater than 5.0 g. The threshold values were based on the Fatty Acid Composition Table from the Standard Tables of Food Composition in Japan (8th Revised Edition), Updated and Enlarged Version 2023 [65].

**Company A (No. 1–30)**		**Chemical Analysis**		
		**ChemL = 1**	**ChemL = 2**	**ChemL = 3**
Tail-cutting	TailL = 1	10	0	0
	TailL = 2	2	4	4
	TailL = 3	0	3	7
**Company B (No. 31–57)**		**Chemical Analysis**		
		**ChemL = 1**	**ChemL = 2**	**ChemL = 3**
Tail-cutting	TailL = 1	0	3	6
	TailL = 2	0	5	4
	TailL = 3	0	0	9
**Both Companies (No. 1–57)**		**Chemical Analysis**		
		**ChemL = 1**	**ChemL = 2**	**ChemL = 3**
Tail-cutting	TailL = 1	10	6	3
	TailL = 2	2	9	8
	TailL = 3	3	3	16

**Table 4:** Mean accuracy [%] of machine learning algorithms and tail-cutting. Each line shows the number of signals used for machine learning input.

	Tail-Cutting	Linear SVM	RBF SVM	LDA	QDA	Random Forest	AdaBoost	MLP	*k*-NN
(a) ChemL = 1 versus ChemL = 2, 3									
1 signal	73.6	59.6	64.0	62.4	66.4	69.3	63.4	62.4	54.7
12 signals	73.6	67.1	73.7	71.1	71.1	81.6	77.6	68.4	53.9
24 signals	73.6	68.4	73.7	71.1	71.1	84.2	81.6	68.4	55.3
(b) ChemL = 1, 2 versus ChemL = 3									
1 signal	78.9	80.0	78.9	77.7	73.7	80.9	75.7	75.4	75.1
12 signals	78.9	81.6	78.9	78.9	71.1	84.2	78.9	81.6	78.9
24 signals	78.9	81.6	78.9	81.6	68.4	84.2	81.6	78.9	78.9

## Data Availability

The original contributions presented in the study are included in the article, further inquiries can be directed to the corresponding author.

## Abbreviations

The following abbreviations are used in this manuscript:

TailL	Tail-cutting label
ChemL	Chemical analysis label
FFT	Fast Fourier transform
SVM	Support vector machine
RBF	Radial basis function
LDA	Linear discriminant analysis
QDA	Quadratic discriminant analysis
AdaBoost	Adaptive boosting
MLP	Multilayer perceptron
*k*-NN	*k*-nearest neighbor
SD	Standard deviation

## Appendix A. Details of Sample

The frozen albacore samples used in this study are listed in Table A1. Each sample was assigned a serial number that corresponds to the numbers shown in the tables and figures in the main text. Table A2 shows statistics of the fat content values corresponding to each tail-cutting label for albacore. Figure A1 shows the tail-cutting surfaces of the frozen albacore samples.

**Table A1:** Detailed sample data and individual information.

Serial Number	TailL	Company	Date	Area
1	1	A	July–September 2022	Atlantic Ocean
2	1	A	July–September 2022	Atlantic Ocean
3	1	A	July–September 2022	Atlantic Ocean
4	1	A	July–September 2022	Atlantic Ocean
5	1	A	July–September 2022	Atlantic Ocean
6	1	A	July–September 2022	Atlantic Ocean
7	1	A	July–September 2022	Atlantic Ocean
8	1	A	July–September 2022	Indian Ocean
9	1	A	July–September 2022	Indian Ocean
10	1	A	July–September 2022	Indian Ocean
11	2	A	July–September 2022	Indian Ocean
12	2	A	July–September 2022	Indian Ocean
13	2	A	July–September 2022	Indian Ocean
14	2	A	July–September 2022	Indian Ocean
15	2	A	July–September 2022	Indian Ocean
16	2	A	July–September 2022	Indian Ocean
17	2	A	July–September 2022	Indian Ocean
18	2	A	July–September 2022	Indian Ocean
19	2	A	July–September 2022	Indian Ocean
20	2	A	July–September 2022	Indian Ocean
21	3	A	July–September 2022	Indian Ocean
22	3	A	July–September 2022	Indian Ocean
23	3	A	July–September 2022	Indian Ocean
24	3	A	July–September 2022	Indian Ocean
25	3	A	July–September 2022	Indian Ocean
26	3	A	July–September 2022	Indian Ocean
27	3	A	July–September 2022	Indian Ocean
28	3	A	July–September 2022	Indian Ocean
29	3	A	July–September 2022	Indian Ocean
30	3	A	July–September 2022	Indian Ocean
31	1	B	19–31 May 2022	Indian Ocean
32	1	B	19–31 May 2022	Indian Ocean
33	1	B	19–31 May 2022	Indian Ocean
34	1	B	19–31 May 2022	Indian Ocean
35	1	B	19–31 May 2022	Indian Ocean
36	1	B	19–31 May 2022	Indian Ocean
37	1	B	19–31 May 2022	Indian Ocean
38	1	B	19–31 May 2022	Indian Ocean
39	1	B	19–31 May 2022	Indian Ocean
40	2	B	19–31 May 2022	Indian Ocean
41	2	B	19–31 May 2022	Indian Ocean
42	2	B	19–31 May 2022	Indian Ocean
43	2	B	19–31 May 2022	Indian Ocean
44	2	B	19–31 May 2022	Indian Ocean
45	2	B	19–31 May 2022	Indian Ocean
46	2	B	19–31 May 2022	Indian Ocean
47	2	B	19–31 May 2022	Indian Ocean
48	2	B	19–31 May 2022	Indian Ocean
49	3	B	19–31 May 2022	Indian Ocean
50	3	B	19–31 May 2022	Indian Ocean
51	3	B	19–31 May 2022	Indian Ocean
52	3	B	19–31 May 2022	Indian Ocean
53	3	B	19–31 May 2022	Indian Ocean
54	3	B	19–31 May 2022	Indian Ocean
55	3	B	19–31 May 2022	Indian Ocean
56	3	B	19–31 May 2022	Indian Ocean
57	3	B	19–31 May 2022	Indian Ocean

**Table A2:** Statistics of the fat content [g/100 g] of each TailL (tail-cutting label).

	Company A (No. 1–30)			Company B (No. 31–57)			Both Companies (No. 1–57)		
	TailL = 1 (N = 10)	TailL = 2 (N = 10)	TailL = 3 (N = 10)	TailL = 1 (N = 9)	TailL = 2 (N = 9)	TailL = 3 (N = 9)	TailL = 1 (N = 19)	TailL = 2 (N = 19)	TaiL = 3 (N = 19)
Maximum	0.0	0.3	1.5	1.4	1.6	6.9	0.0	0.3	1.5
Minimum	0.3	9.4	13.5	7.8	12.6	13.9	7.8	12.6	13.9
Mean	0.1	3.6	8.4	4.0	5.1	10.0	2.0	4.3	9.1
SD	0.1	1.3	3.7	2.0	3.5	2.4	2.4	3.2	3.3
